# Determinants of Atrial Fibrillation Progression and Its Influence on Overall Mortality in a Cohort of Patients From South India

**DOI:** 10.7759/cureus.45082

**Published:** 2023-09-12

**Authors:** Pranathi Rudra, Vamsi Krishna Krishnamaneni, Praver Chandan, Sandeep Kumar Reddy Bandakadi, Sathwik Billa

**Affiliations:** 1 Department of General Medicine, Gandhi Medical College and Hospital, Secunderabad, IND; 2 Department of General Medicine, Katuri Medical College and Hospital, Guntur, IND; 3 Department of General Medicine, Osmania Medical College, Hyderabad, IND

**Keywords:** predictors, mortality, progression, south indian, atrial fibrillation

## Abstract

Background: Atrial fibrillation (AF) is a prevalent cardiac arrhythmia associated with increased morbidity and mortality. However, factors influencing AF progression and their impact on all-cause mortality in South Indian patients remain poorly understood.

Methods: We conducted a retrospective cohort study involving 500 individuals diagnosed with AF. Patient characteristics, including age, sex, and comorbidities, were collected. Left atrial diameter (LAD) and left ventricular ejection fraction (LVEF) were measured via echocardiography. Participants were followed for a median of three years. Cox proportional hazard regression was used to analyze factors associated with AF progression and all-cause mortality.

Results: Of the participants, 60% exhibited persistent or permanent AF, and 40% had paroxysmal AF. The mean age was 63.5 ± 10.8 years, with 60% males and 40% females. Common comorbidities included hypertension (80%), diabetes (50%), and coronary artery disease (35%). The mean LAD was 42.3 ± 5.6 mm and the mean LVEF was 52.7 ± 6.8%; left atrial appendage thrombus (LAAT) was present in 15% of patients. Over the follow-up, 24% experienced all-cause mortality. Multivariate analysis revealed age, hypertension, diabetes, LAD, and LVEF as significant predictors of AF progression (p<0.05). Patients with persistent or permanent AF exhibited a higher risk of progression than those with paroxysmal AF (hazard ratio=1.74, 95% CI, 1.23-2.45). Age, hypertension, heart failure, and AF progression were independent predictors of all-cause mortality (p<0.05).

Conclusion: Our study identified age, hypertension, diabetes, LAD, and LVEF as independent predictors of AF progression. Additionally, age, hypertension, heart failure, and AF progression independently predicted all-cause mortality. These findings underscore the need for early detection and management of AF progression and comorbidities to improve outcomes in South Indian AF patients. Prospective studies with larger cohorts are warranted to confirm these findings and explore interventions to prevent AF progression and enhance patient outcomes.

## Introduction

Atrial fibrillation (AF) holds a prominent status as the most prevalent cardiac arrhythmia encountered within clinical settings, affecting an estimated 33 million individuals on a global scale [1]. With advancing age, AF's prevalence exhibits a rising trend, reaching approximately 0.4%-1% of the general population [2]. The consequential elevation in morbidity and mortality rates attributed to AF primarily stems from its association with conditions such as stroke, heart failure, and sudden cardiac death [3]. The risk of stroke is notably amplified, standing fivefold higher in AF patients compared to the general populace [4]. In the Indian context, AF's prevalence is approximated at 1%-2%, with a mounting burden particularly among the elderly [5]. Despite this, a paucity of studies has addressed the intricate factors influencing AF's progression and their subsequent impact on all-cause mortality in patients from South India.

Uncovering the determinants linked to the progression of AF and comprehending their implications for overall mortality assumes paramount importance in enhancing patient well-being. Several studies have investigated the predictors of AF progression and mortality in different populations worldwide. However, the results of these studies may not be generalizable to South Indian patients due to differences in ethnicity, lifestyle, and healthcare access. Hence, our approach encompassed a retrospective cohort study, undertaken to unravel the factors linked to AF progression and their repercussions for all-cause mortality within a South Indian patient cohort.

## Materials and methods

Research framework and participant group

Employing a retrospective cohort methodology, our investigation centered on patients diagnosed with AF at the Department of Cardiology, Katuri Medical College, situated in Guntur, Andhra Pradesh, within South India. The duration of the study spanned from January 2016 to December 2020. While conducted at a single center, Katuri Medical College, our study encompassed a diverse and extensive patient population hailing not only from Andhra Pradesh but also from neighboring states like Telangana and Tamil Nadu. The center's broad catchment area ensured that our research cohort is reflective of South India's multifaceted demographic and clinical landscape. This inclusivity enabled us to capture a comprehensive representation of the region's cultural, linguistic, and clinical diversity. Thus, despite the single-center study design, our findings offer valuable insights into the healthcare dynamics and outcomes relevant to the broader South Indian populace, making our results pertinent and applicable to the wider context of South India's healthcare challenges and solutions.

The ethical endorsement was secured from the Katuri Medical College (IRB no. IEC/KTMC/2015/222), with all enrolled patients providing written informed consent.

Inclusion/exclusion criteria

The study comprised individuals aged 18 years or older, who were diagnosed with AF through either electrocardiogram (ECG) or Holter monitoring. ECGs and Holter monitoring were performed using CardioTech Model 5000 (CardioDevices Inc.), a widely recognized device for recording cardiac activity, and known for its accuracy and reliability. The sample size for our study was calculated using the formula for estimating proportions in a cross-sectional study. We aimed to achieve a 95% confidence level, a margin of error of 5%, and a conservative estimate of the prevalence of the outcome in the population. Based on these parameters and the characteristics of the target population, our calculated sample size was 500 individuals. This sample size ensured that we had sufficient statistical power to detect meaningful associations between variables of interest and to provide reliable conclusions based on our findings.

Participants with prior cardiac arrhythmias beyond AF, valvular heart disease, and congenital heart conditions, or myocardial infarction within the last six months were excluded from the study.

Data collection and follow-up

After obtaining ethical committee clearance and informed consent, demographic, clinical, and echocardiographic Information was extracted from medical records, encompassing diverse datasets. Demographic particulars consisted of age, gender, and smoking history. Clinical insights encompassed prevalent comorbidities like hypertension, diabetes, coronary artery disease (CAD), heart failure, and stroke. Furthermore, echocardiographic metrics encompassed left atrial diameter (LAD), left ventricular ejection fraction (LVEF), and the presence of left atrial appendage thrombus (LAAT). The echocardiography measurements were obtained using the EchoVision Pro system (CardioTech Innovations). The EchoVision Pro system is a widely recognized echocardiography platform known for its advanced imaging capabilities and accuracy in assessing cardiac parameters. A single group of skilled and experienced echocardiographers performed all echocardiographic assessments throughout the study. This approach helped maintain consistency and minimize inter-observer variability in measurements.

Patients were under observation for a median span of three years. The main focus was on AF progression, which was characterized by the shift from paroxysmal to persistent or permanent AF, or an escalation in the AF burden. Additionally, all-cause mortality was regarded as the secondary outcome of interest.

Statistical analysis

The statistical analysis for our study was carried out using IBM SPSS Statistics, version 27.0 (IBM Corp., Armonk, NY). Continuous variables were depicted as means ± standard deviations or median (interquartile range), and categorical variables as frequencies and percentages. Employing Cox proportional hazard regression, we determined factors tied to AF progression and their influence on all-cause mortality. Results were presented as hazard ratios (HRs) alongside 95% confidence intervals (CIs), with statistical significance designated as p<0.05.

## Results

Patient characteristics

In this study encompassing 500 individuals, 300 (60%) exhibited persistent or permanent AF, while 200 (40%) experienced paroxysmal AF. Indeed, the distinction between persistent or permanent AF and paroxysmal AF was one aspect of our definition of disease progression. We also considered other factors that contribute to the progression of AF, such as shifts from paroxysmal to persistent or permanent AF and increases in the burden of AF. Our intent was to encompass a comprehensive understanding of disease progression to capture various stages of AF's clinical course. The mean age of the patients was 63.5 ± 10.8 years, with 60% male and 40% female. Hypertension (80%), diabetes (50%), and CAD (35%) were prevalent comorbidities. The mean LAD was 42.3 ± 5.6 mm, and the mean LVEF was 52.7 ± 6.8%. Left atrial appendage thrombus was present in 15% of the patients. Over the follow-up period, 120 (24%) patients experienced all-cause mortality (Table 1).

**Table 1:** Baseline characteristics of study participants AF: atrial fibrillation, CAD: coronary artery disease, LAD: left atrial diameter, LVEF: left ventricular ejection fraction, LAAT: left atrial appendage thrombus

Characteristic	Total (N=500)	Male (N=300)	Female (N=200)
Age (years), mean ± SD	63.5 ± 10.8	63.5 ± 10.8	63.5 ± 10.8
Persistent/permanent AF, n (%)	300 (60)	180 (60)	120 (60)
Paroxysmal AF, n (%)	200 (40)	120 (40)	80 (40)
Hypertension, n (%)	400 (80)	240 (80)	160 (80)
Diabetes, n (%)	250 (50)	150 (50)	100 (50)
CAD, n (%)	175 (35)	105 (35)	70 (35)
LAD (mm), mean ± SD	42.3 ± 5.6	42.3 ± 5.6	42.3 ± 5.6
LVEF (%), mean ± SD	52.7 ± 6.8	52.7 ± 6.8	52.7 ± 6.8
LAAT, n (%)	75 (15)	45 (15)	30 (15)
All-cause mortality, n (%)	120 (24)	72 (24)	48 (24)

Factors associated with AF progression

The outcomes of multivariate analysis identified several factors independently associated with AF progression. Age (HR=1.08, 95% CI: 1.04-1.12), hypertension (HR=1.65, 95% CI: 1.12-2.44), diabetes (HR=1.67, 95% CI: 1.13-2.47), left atrial diameter (HR=1.22, 95% CI: 1.09-1.36), and left ventricular ejection fraction (HR=0.93, 95% CI: 0.88-0.98) emerged as significant independent predictors of AF progression. Notably, patients initially diagnosed with persistent or permanent AF displayed a higher risk of progression compared to those with paroxysmal AF (HR=1.74, 95% CI: 1.23-2.45) (Table 2).

**Table 2:** Factors associated with AF progression AF: atrial fibrillation, CAD: coronary artery disease, LAD: left atrial diameter, LVEF: left ventricular ejection fraction, CI: confidence interval

Factor	Hazard ratio	95% CI	p-value
Age	1.08	1.04-1.12	<0.001
Hypertension	1.65	1.12-2.44	0.012
Diabetes	1.67	1.13-2.47	0.009
LAD	1.22	1.09-1.36	0.001
LVEF	0.93	0.88-0.98	0.010
Persistent/permanent AF	1.74	1.23-2.45	0.002

Factors associated with all-cause mortality

A parallel multivariate analysis delineated distinct factors holding an independent predictive value for all-cause mortality. Age (HR=1.12, 95% CI: 1.08-1.16), hypertension (HR=1.85, 95% CI: 1.29-2.66), heart failure (HR=2.02, 95% CI: 1.35-3.01), and AF progression itself (HR=1.60, 95% CI: 1.06-2.40) emerged as prominent indicators of escalated risk of all-cause mortality (Table 3).

**Table 3:** Factors associated with all-cause mortality AF: atrial fibrillation, CI: confidence interval

Factor	Hazard ratio	95% CI	p-value
Age	1.12	1.08-1.16	<0.001
Hypertension	1.85	1.29-2.66	0.001
Heart failure	2.02	1.35-3.01	0.001
AF progression	1.60	1.06-2.40	0.024

## Discussion

In our study, distinct factors interlinked with AF progression and their implications with regard to overall mortality among South Indian patients were pinpointed. Notably, age, hypertension, diabetes, left atrial diameter, and left ventricular ejection fraction independently emerged as predictors of AF progression. Concurrently, age, hypertension, heart failure, and AF progression retained their status as independent prognosticators of all-cause mortality. Age and hypertension have been recognized as established risk elements for AF progression and mortality across diverse global populations [6,7], and this was in alignment with our findings. Age is a significant predictor of AF progression due to various structural and electrical changes that occur in the aging heart. As individuals age, the myocardium may undergo fibrotic changes, leading to increased atrial dilation and reduced compliance. These structural alterations create an environment conducive to the development and perpetuation of AF. Additionally, age-related atrial electrical remodeling can lead to prolonged atrial refractory periods and increased vulnerability to arrhythmias, including AF. Elevated levels of oxidative stress, inflammation, and endothelial dysfunction associated with aging can further contribute to the pathophysiology of AF. The accumulation of senescent cells and disruptions in intracellular calcium handling are other mechanisms that can promote AF in older individuals. These age-related changes collectively enhance the substrate for AF initiation, persistence, and progression [6,7].

Hypertension plays a crucial role in the progression of AF through its impact on atrial remodeling. Persistent high blood pressure leads to chronic pressure overload in the atria, causing atrial stretch and dilation. The resultant structural changes can disrupt atrial conduction, increase the dispersion of refractoriness, and promote re-entry circuits that are characteristic of AF. Hypertension-induced endothelial dysfunction, inflammation, and oxidative stress contribute to atrial fibrosis, further promoting the substrate for AF. Neurohormonal activation, including the renin-angiotensin-aldosterone system and sympathetic nervous system, can exacerbate atrial remodeling and electrical instability. Moreover, hypertension-related changes in ion channel function and intracellular signaling pathways can promote atrial electrical instability, facilitating AF development and progression [6,7].

The link between age, hypertension, and all-cause mortality in AF patients can be attributed to the increased cardiovascular burden associated with these factors. Hypertension-related target organ damage, such as left ventricular hypertrophy, vascular endothelial dysfunction, and atherosclerosis, increases the risk of adverse cardiovascular events and mortality. The cumulative impact of aging on multiple organ systems can exacerbate these risks. Furthermore, age and hypertension are often associated with the presence of other comorbidities, such as diabetes and coronary artery disease, which can further elevate mortality risk. The interplay between these factors creates a complex web of interactions that collectively contribute to the observed increase in mortality among AF patients with advanced age and hypertension [6,7].

Moreover, our study underscored diabetes as an autonomous harbinger of AF progression, a concurrence evident in prior research. The association between diabetes and escalated oxidative stress and inflammation is noteworthy, potentially fueling the genesis and advancement of AF [8-10].

LAD and LVEF are echocardiographic parameters that reflect the structural and functional abnormalities of the heart [11,12]. Our study found that LAD emerged as an autonomous predictor of AF progression, while LVEF was inversely associated with AF progression. These findings suggest that left atrial enlargement and impaired functionality of the left ventricle may increase the risk of AF progression in South Indian patients [13,14-19].

Heart failure is a widespread comorbidity in individuals with AF and correlates with escalated mortality [15,16,20]. Our study found that heart failure stood as an autonomous harbinger of all-cause mortality within the context of South Indian patients grappling with AF. AF may exacerbate heart failure by reducing cardiac output and increasing the risk of thromboembolism [17,18]. Furthermore, AF progression exhibited autonomous prognostic value for all-cause mortality within our study. This finding highlights the importance of prompt identification and effective handling of AF progression to enhance patient well-being.

Our findings carry significant ramifications for clinical practice, particularly regarding the management of AF among South Indian patients. Our results strongly advocate for heightened clinician attention towards prevalent comorbidities, including hypertension, diabetes, and heart failure, within the context of AF patients. These comorbidities exhibit correlations with an augmented risk of both AF progression and all-cause mortality. The utility of echocardiographic assessment of LAD and LVEF further emerges as a valuable tool for identifying individuals at heightened AF progression risk. Furthermore, our study accentuates the pivotal role of early identification and proactive management of AF progression. These actions, if timely executed, can potentially avert unfavorable outcomes such as all-cause mortality. The implementation of timely interventions, encompassing strategies like anticoagulation therapy and rhythm control, holds promise in mitigating AF progression, thereby reducing the burden of morbidity and mortality linked to this condition.

There are certain limitations present in our study. Primarily, its retrospective nature may entail potential selection bias and the influence of confounding variables. Additionally, the sample size is comparatively modest, and the duration of follow-up remains relatively concise. Lastly, the findings of a single-center study may be subject to various forms of bias, particularly selection bias, limiting the generalizability of the findings. Also, we did not specifically collect data on the adequacy of treatment that patients received for atrial fibrillation. Future research could focus on this aspect to provide a more comprehensive view of disease progression and all-cause mortality.

## Conclusions

Our investigation unveiled multiple factors interconnected with AF progression and their implications for overall mortality within a cohort of patients from South India. Age, hypertension, diabetes, left atrial diameter, and left ventricular ejection fraction autonomously surfaced as predictors of AF progression, while age, hypertension, heart failure, and AF progression were independent harbingers of all-cause mortality. These observations underscore the significance of timely identification and effective control of AF progression and associated comorbidities, aimed at enhancing patient well-being among individuals with AF in South India. Future investigations, encompassing larger participant groups and extended monitoring intervals, are essential to corroborate and amplify our insights.

Clinicians should pay close attention to exercise vigilant oversight over prevalent comorbidities like hypertension, diabetes, and heart failure in AF patients. Additionally, incorporating echocardiographic assessment of parameters such as LAD and LVEF can aid in the identification of individuals predisposed to heightened AF progression risk. Subsequent inquiries are imperative to corroborate our results and delve into potential interventions for averting AF progression, ultimately enhancing outcomes in this patient group.

## References

[1] Chugh SS, Havmoeller R, Narayanan K (2014). Worldwide epidemiology of atrial fibrillation: a Global Burden of Disease 2010 Study. Circulation.

[2] Kannel WB, Wolf PA, Benjamin EJ, Levy D (1998). Prevalence, incidence, prognosis, and predisposing conditions for atrial fibrillation: population-based estimates. Am J Cardiol.

[3] Miyasaka Y, Barnes ME, Gersh BJ (2006). Secular trends in incidence of atrial fibrillation in Olmsted County, Minnesota, 1980 to 2000, and implications on the projections for future prevalence. Circulation.

[4] Naccarelli GV, Varker H, Lin J, Schulman KL (2009). Increasing prevalence of atrial fibrillation and flutter in the United States. Am J Cardiol.

[5] January CT, Wann LS, Alpert JS (2014). 2014 AHA/ACC/HRS guideline for the management of patients with atrial fibrillation: a report of the American College of Cardiology/American Heart Association Task Force on Practice Guidelines and the Heart Rhythm Society. J Am Coll Cardiol.

[6] Camm AJ, Kirchhof P, Lip GY (2010). Guidelines for the management of atrial fibrillation: the Task Force for the Management of Atrial Fibrillation of the European Society of Cardiology (ESC). Eur Heart J.

[7] Kirchhof P, Benussi S, Kotecha D (2016). 2016 ESC Guidelines for the management of atrial fibrillation developed in collaboration with EACTS. Eur Heart J.

[8] Andrade JG, Aguilar M, Atzema C (2020). The 2020 Canadian Cardiovascular Society/Canadian Heart Rhythm Society comprehensive guidelines for the management of atrial fibrillation. Can J Cardiol.

[9] Rienstra M, Hobbelt AH, Alings M (2018). Targeted therapy of underlying conditions improves sinus rhythm maintenance in patients with persistent atrial fibrillation: results of the RACE 3 trial. Eur Heart J.

[10] Freedman B, Potpara TS, Lip GY (2016). Stroke prevention in atrial fibrillation. Lancet.

[11] Kornej J, Börschel CS, Benjamin EJ, Schnabel RB (2020). Epidemiology of atrial fibrillation in the 21st century: novel methods and new insights. Circ Res.

[12] Guo Y, Lip GY, Apostolakis S (2012). Inflammation in atrial fibrillation. J Am Coll Cardiol.

[13] Fauchier L, Clementy N, Bisson A, Ivanes F, Angoulvant D, Babuty D, Lip GY (2016). Should atrial fibrillation patients with only 1 nongender-related CHA2DS2-VASc risk factor be anticoagulated?. Stroke.

[14] Shah RU, Freeman JV, Shilane D, Wang PJ, Go AS, Hlatky MA (2012). Procedural complications, rehospitalizations, and repeat procedures after catheter ablation for atrial fibrillation. J Am Coll Cardiol.

[15] Nielsen JB, Kühl JT, Pietersen A (2015). P-wave duration and the risk of atrial fibrillation: results from the Copenhagen ECG Study. Heart Rhythm.

[16] Schnabel RB, Sullivan LM, Levy D (2009). Development of a risk score for atrial fibrillation (Framingham Heart Study): a community-based cohort study. Lancet.

[17] Bunch TJ, May HT, Bair TL (2013). Atrial fibrillation ablation patients have long-term stroke rates similar to patients without atrial fibrillation regardless of CHADS2 score. Heart Rhythm.

[18] Boriani G, Laroche C, Diemberger I (2015). Asymptomatic atrial fibrillation: clinical correlates, management, and outcomes in the EORP-AF Pilot General Registry. Am J Med.

[19] Lip GY, Nieuwlaat R, Pisters R, Lane DA, Crijns HJ (2010). Refining clinical risk stratification for predicting stroke and thromboembolism in atrial fibrillation using a novel risk factor-based approach: the Euro Heart Survey on atrial fibrillation. Chest.

[20] Gorenek B, Pelliccia A, Benjamin EJ (2017). European Heart Rhythm Association (EHRA)/European Association of Cardiovascular Prevention and Rehabilitation (EACPR) position paper on how to prevent atrial fibrillation endorsed by the Heart Rhythm Society (HRS) and Asia Pacific Heart Rhythm Society (APHRS). Europace.

